# Complement regulatory protein CD46 induces autophagy against oxidative stress-mediated apoptosis in normal and asthmatic airway epithelium

**DOI:** 10.1038/s41598-018-31317-5

**Published:** 2018-08-28

**Authors:** Yi-Giien Tsai, Yung-Sung Wen, Jiu-Yao Wang, Kuender D. Yang, Hai-Lun Sun, Jia-Hung Liou, Ching-Yuang Lin

**Affiliations:** 1 Department of Pediatrics, Changhua Christian Children’s Hospital, Changhua, Taiwan; 2 School of Medicine, Kaohsiung Medical University, Kaohsiung, Taiwan; 3 School of Medicine, Chung Shan Medical University, Taichung, Taiwan; 4 Department of Otorhinolaryngology, Head and Neck Surgery, Changhua Christian Hospital, Changhua, Taiwan; 5 Department of Pediatrics, College of Medicine, National Cheng Kung University Hospital, Tainan, Taiwan; 6 Mackay Children’s Hospital, and Institute of Biomedical Sciences, Mackay Medical College, Taipei, Taiwan; 7 Department of Pathology, Changhua Christian Hospital, Changhua, Taiwan; 8 Clinical Immunological Center and College of Medicine, China Medical University Hospital, Taichung, Taiwan

## Abstract

Autophagy plays a major role in defending against oxidative stress in respiratory epithelial cells. The complement regulatory protein CD46 can enhance autophagy and decrease local complement activation at sites of inflammation. This study investigated the mechanism by which CD46 protects against oxidative stress-mediated apoptosis in respiratory epithelium in asthmatic patients. Nasal mucosa samples were obtained from 60 adults with mild asthma who received turbinectomy and 30 controls. A decreased expression of CD46 and increased apoptosis were noted in the damaged nasal epithelium from the asthmatic patients. Primary epithelial cells cultured with *Dermatophagoides pteronyssinus* 2 showed decreased CD46 and increased cleaved CASPASE-3A expressions. Crosslinking CD46 mAb could induce the formation of autophagosomes and LC3-II expression in primary epithelial cells. CD46 engagement could induce autophagy against hydrogen peroxide-induced epithelial cell death, whereas the autophagy inhibitor 3-methyladenine decreased this effect. In addition, CD46 engagement decreased the expressions of PRO-IL-1β and NLRP3, enhanced the expression of scaffold protein GOPC, and diminished hydrogen peroxide-induced 8-OHdG, IL-1β and IL-6 production. Silencing *ATG5* in human lung epithelial A549 cells decreased CD46-activated autophagy with LC3-II. CD46 induced autophagy and decreased the oxidative stress-mediated apoptosis of respiratory epithelium, and this may offer a new therapeutic strategy to treat asthma.

## Introduction

The bronchial epithelium plays an important role in chronic airway inflammation, bronchial hyperreactivity and airway wall remodeling in allergic asthma^1,2^. The respiratory epithelium forms an interface with the external environment and can be damaged by oxidative stress^3,4^. Numerous studies have reported increased levels of reactive oxygen species (ROS) and decreased levels of antioxidants in asthmatic patients^5–7^. The susceptibility of airway epithelial cells to oxidative stress has been shown to increases with allergic sensitization, and exposure to allergens or environmental pollutant has been shown to increase airway inflammation^8–10^. Bronchial epithelial cells that produce proinflammatory signals in response to ROS may worsen the airway response and have been associated to the severity of asthma^11–13^.

Normal bronchial epithelial cells are relatively refractory to apoptotic stimulation when exposed to ROS and death receptor ligands secreted by inflammatory cells^14^. However, abnormal apoptotic mechanisms which disrupt the bronchial epithelial barrier have been associated with the pathogenesis of asthma. Moreover, excess oxidative stress has been reported to result in chromatin dysfunction, apoptosis and necrosis with loss of columnar epithelial cells in asthma^14–16^.

Autophagy is an intracellular degradation mechanism that eliminates damaged organelles and promotes survival during starvation^17,18^. Accumulating evidence suggests that autophagy can modulate cellular death, inflammation and immune function^17–19^, and that impaired autophagy may lead to accelerated senescence, neurodegenerative diseases, cancer and inflammatory bowel disease^20–23^. The integrity of the epithelial barrier depends on homeostatic regulatory mechanisms, and autophagy may protect against oxidative stress in respiratory diseases^24–28^.

The complement system has been reported to be locally and systemically activated to amplify inflammatory responses in allergic asthma^29,30^. The complement regulatory protein CD46 is widely distributed in human leukocytes, epithelial cells and fibroblasts, and it has been shown to have a protective effect against autologous complement-mediated lysis at sites of inflammation^31,32^. Complement regulatory proteins may interfere with oxidative stress-programmed apoptosis to avoid triggering inflammation. In addition, surface CD46 has been shown to be rapidly lost from apoptotic T cells to facilitate their rapid complement-mediated removal^33^. Crosslinking CD46 during T-cell receptor activation has been shown to lead to the development of inducible T regulatory cells^34–36^, which may assist in maintaining immune tolerance in autoimmune diseases^37^ and allergic asthma^35,36^.

A high expressions of CD46 in chronic obstructive pulmonary diseases has been reported to protect against lung inflammation by T regulatory cells and restraining complement cascade-induced apoptosis^38^. Autophagy is important for innate cellular defense against viral and bacterial pathogens. Two CD46-binding pathogens, measles virus and group A Streptococcus, have been shown to induce autophagy pathways^39,40^. Targeting autophagy and apoptosis manipulating factors in inflamed respiratory epithelium is important to decrease ongoing damage in respiratory epithelium and consequent airway remodeling. In this study, we assessed the functional role of CD46 in respiratory epithelium with regards to autophagy and apoptosis in asthmatic patients. Our findings may provide further evidence regarding the practical application of CD46 in clinical practice to protect respiratory epithelium in patients with asthma.

## Results

### Decreased Expression of CD46 and Increased Apoptosis in the Damaged Nasal Epithelium of the Asthmatic Patients

**Figure 1:**
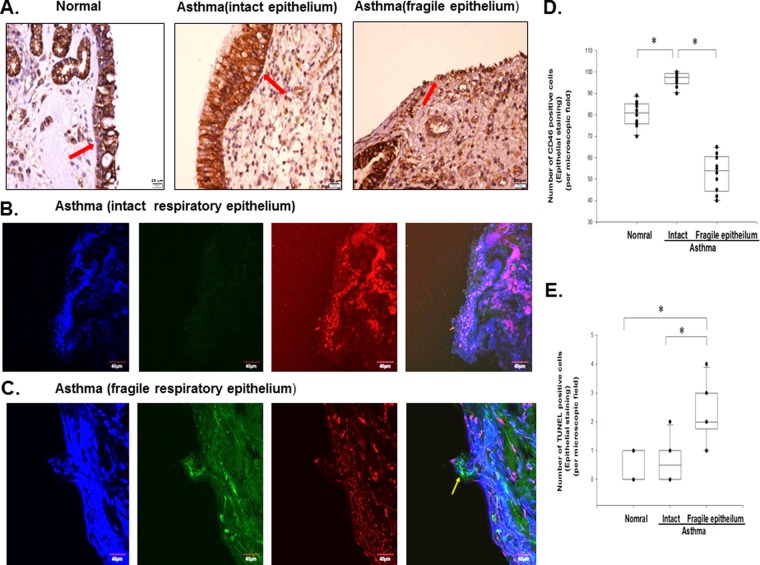
Increased apoptosis and decreased CD46 in the nasal mucosa of the asthmatic patients. (**A**) CD46 expression (red arrow) was increased in the asthmatic patients (n = 10) compared with the healthy controls (n = 10) as shown by immunohistochemical staining. Epithelial cell shedding and a decreased expression of CD46 (red arrow) were noted in the fragile epithelium of the asthmatic patients (n = 10). Representative confocal microscopic analysis between intact nasal epithelium (**B**) and fragile epithelium (**C**) from the asthmatic patients. Primary nasal epithelium biopsy stained with TUNEL (FITC), CD46 (PE), and 4′,6-diamidino-2-phenylindole (DAPI) (nuclear stain). The yellow arrows indicate TUNEL-positive cells. Decreased CD46 expression and increased TUNEL staining in the fragile upper airway epithelial cells are shown. Scale bar, 20 um. (**D**,**E**) Statistical data of 10 paired experiments between intact and fragile epithelium from asthmatic patients for the number of CD46 and TUNEL-positive cells (% epithelial cells stained) per high-power field. The Kruskal-Wallis test was used. *p < 0.05.

The patient characteristics are shown in Table 1. To examine the relationship between CD46 and apoptosis in the respiratory epithelium, we analyzed the expression of CD46 and apoptosis in nasal epithelium samples from the normal controls and asthmatic patients who received nasal polypectomy. The area of intact epithelium of nasal biopsy samples taken from the normal controls showed mild immunoreactivity for CD46 (Fig. 1A). However, intact epithelium from the asthmatic patients showed strong immunostaining for CD46 (red arrow), and a decreased CD46 expression in desquamated nasal epithelium (Fig. 1A). Representative confocal microscopic analysis of the nasal mucosa biopsies between intact nasal epithelium (Fig. 1B) and fragile epithelium (Fig. 1C) from the asthmatic patients were shown. Confocal microscopic analysis of the nasal mucosa biopsies from the asthmatic patients revealed increased immunoreactivity for CD46 without TUNEL staining in intact epithelium (Fig. 1B). TUNEL-positive epithelial cells (yellow arrow) were detected with decreased immunoreactivity for CD46 in fragile epithelium in the asthmatic patients (Fig. 1C). Experiments were performed with 10 paired samples, and the results showed significant statistical differences (*p* < 0.05) (Fig. 1D,E).

**Table 1:** Patient characteristics.

	Asthmatic group	Control group
Number of patients	60	30
Mean age (years)	46.6 ± 16.3	46.4 ± 13.0
Gender (M:F)	46:24	19:11
Der p-specific IgE (kU/l)	66.5 ± 21.1	ND
Mean FEV1 (%)	89.3 ± 7.97	93.5 ± 5.31

ND: Not Detectable.

### Decreased CD46 and Increased *Dermatophagoides pteronyssinus* 2-mediated Cell Death of the Primary Epithelial Cells from the Asthmatic Patients

**Figure 2:**
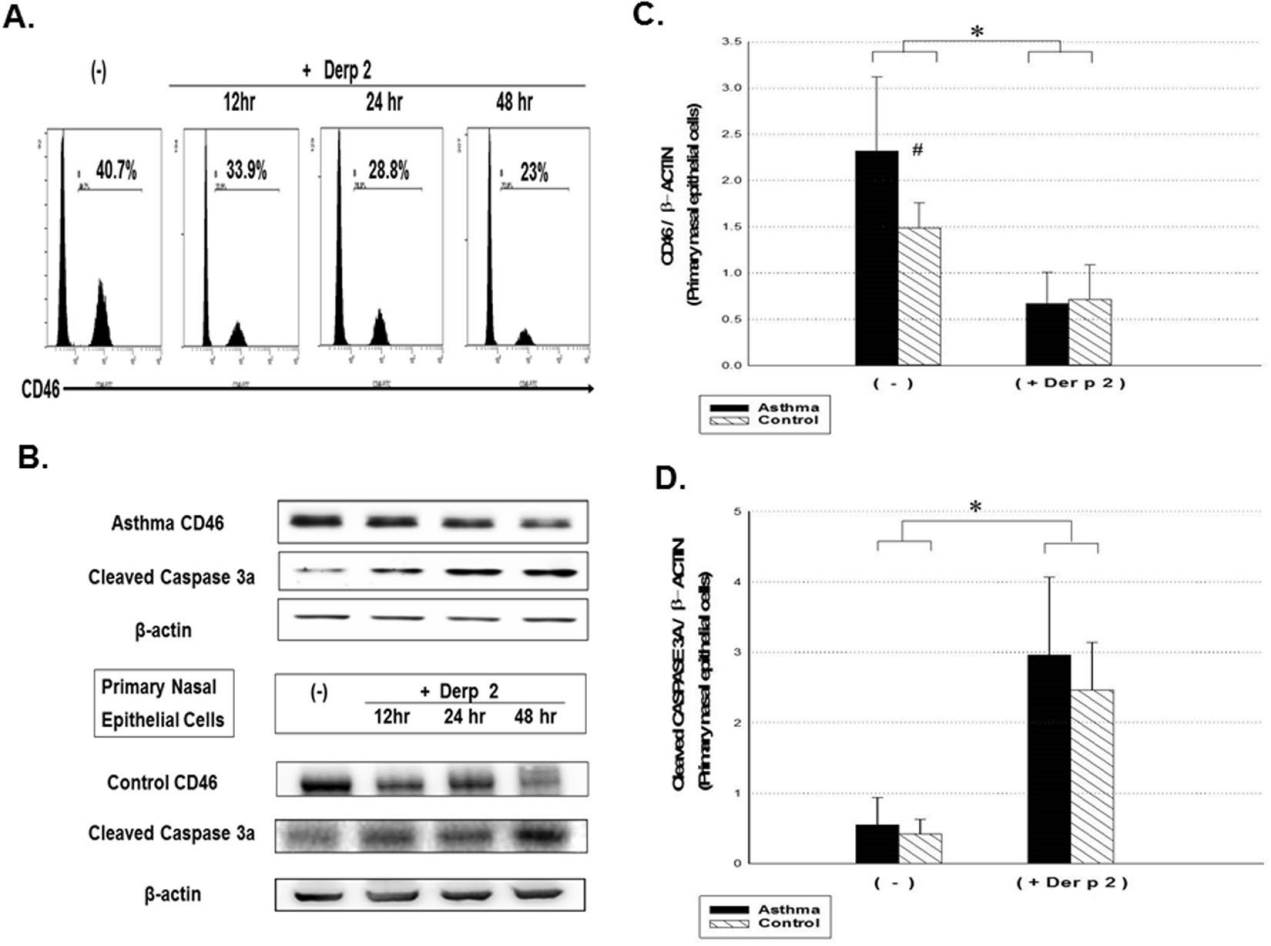
Analysis of CD46 and cleaved CASPASE-3A activity from Der p 2-mediated apoptosis of primary upper airway epithelial cells in the asthmatic patients and control subjects. (**A**) Primary nasal epithelial cells from the asthmatic patients stimulated with Der p 2 (10 μg/ml) and CD46 expression analyzed at 12, 24 and 48 hours by flow cytometry. Representative profiles are shown. (**B**) Western blot analysis was performed to detect CD46 and cleaved CASPASE-3A activity from Der p 2-treated epithelial cells for 48 hours from the asthmatic patients and control subjects. Statistical data of 30 paired experiments between asthmatic patients and control subjects for the expressions of CD46 (**C**) and cleaved CASPASE-3A (**D**) after Der p 2 stimulation for 48 hours. The Kruskal-Wallis test (among multiple groups) and Wilcoxon signed ranked test (after Der p 2) were used. *p < 0.05 after Der p2 and ^#^*p* < 0.05 compared to the control group.

To evaluate the role of CD46 in the pathogenesis of respiratory epithelium apoptosis, we evaluated the CD46 expression with mite allergen-induced epithelial cell death in the asthmatic patients. *Dermatophagoides pteronyssinus* 2 (Der p 2) is the major allergen in Taiwan. It contains a cysteine protease which may cause proteolysis of CD46 with a subsequent increase in the apoptosis of respiratory epithelial cells^35^. After Der p 2 stimulation, the expressions of the apoptosis markers with cleaved CASPASE-3A and CD46 were analyzed in primary nasal epithelial cells from the asthmatic patients. A decrease in the expression of CD46 was noted in the epithelial cells (Fig. 2A), with an increase in the expression of cleaved CASPASE-3A (as shown by Western blotting; Fig. 2B) in the asthmatic patients (*p* < 0.05). Experiments were performed with 30 paired samples, and the statistical results are shown in Fig. 2C,D.

### Autophagy Induced by CD46 Antibody Activation in Normal and Asthmatic Epithelial Cells

**Figure 3:**
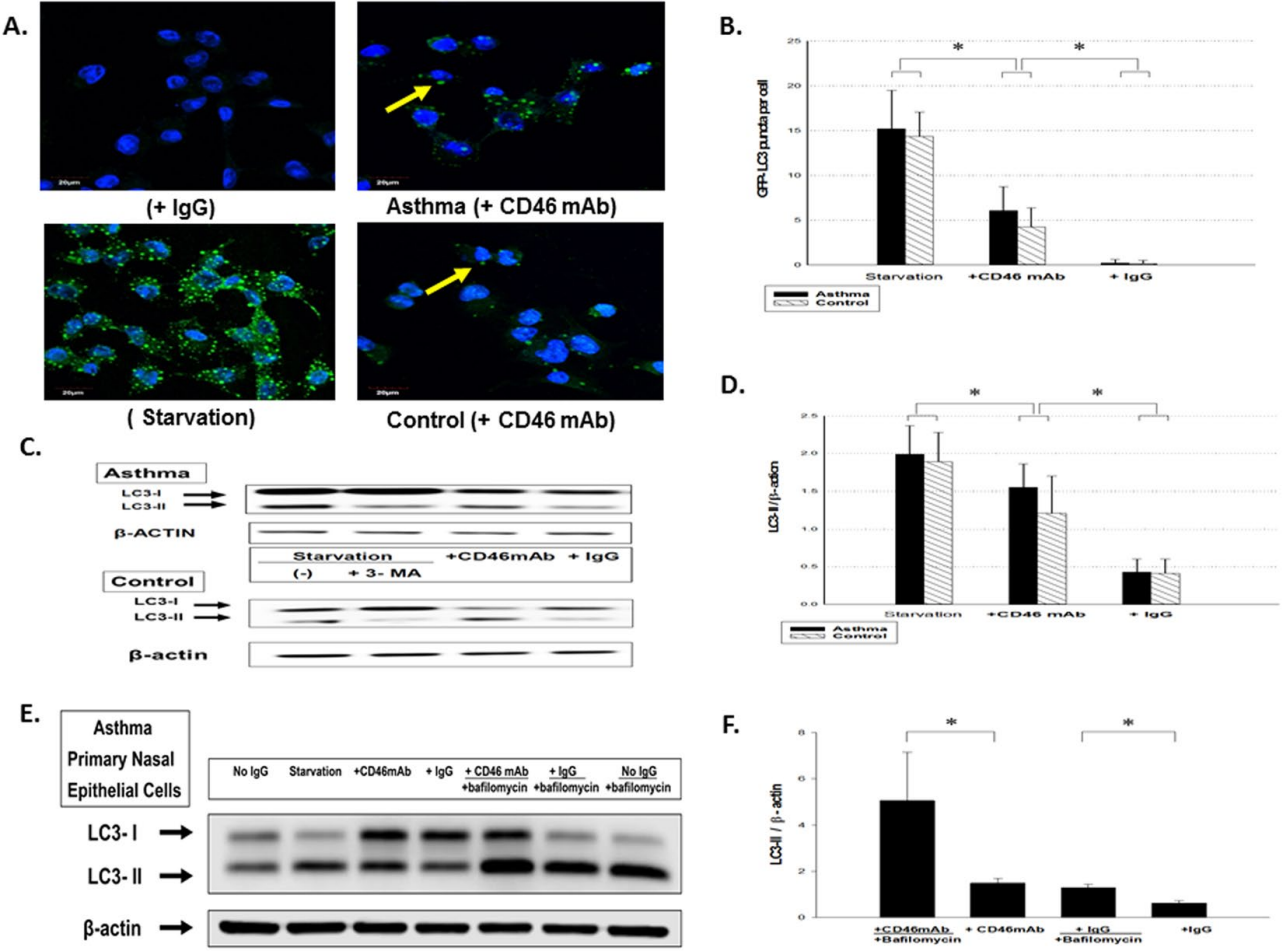
CD46 induced autophagy in primary upper airway epithelial cells from the controls and asthmatic patients. Cells were incubated for 4 hours in complete medium in the presence of anti-CD46 mAb (5 μg/ml), isotype control antibody (IgG), or in nutrient-deprived media (starvation) and/or the autophagy inhibitor 3-methyladenine (3-MA) (10 mmol/L) (Sigma-Aldrich, St. Louis, MO). (**A**) Representative images of GFP-LC3 puncta (autophagosomes) in nasal epithelial cells are shown by confocal microscopy. (**B**) The cytosolic soluble form of LC3-I was converted into the autophagic vesicle-associated form of LC3-II and was used as a marker of autophagosome formation. The number of GFP-LC3 vesicles per cell in primary nasal epithelial cells was calculated from 200 cells for each experiment. Quantification of GFP-LC3 puncta per cell was assayed and the statistical data are shown. (**C**) Immunoblotting was used to analyze the LC3- II expression in primary upper airway epithelial cells from the controls and asthmatic patients. (**D**) Statistical data of the experiments with 30 paired samples as shown. (**E**) Primary nasal epithelial cells from the asthmatic subjects were treated with bafilomycin, and immunoblotting was used to analyze the expressions of LC3-II. (**F**) Statistical data of the experiments with six paired samples as shown. The Kruskal-Wallis test was used to determine significant differences. *p < 0.05.

Engagement of CD46, a ubiquitous human surface receptor able to bind several different pathogens, can induce autophagy to control infection^39^. To further investigate the role of CD46 in the autophagy of respiratory epithelium, we examined whether antibody-driven CD46 crosslinking could induce the formation of autophagosomes. Crosslinking CD46 mAb at the surface of primary nasal airway epithelial cells induced macropinocytosis-like internalization, and led to the degradation of the cell surface of CD46 via the same molecular mechanism as the CD46 ligand (Supplement Fig. 1). CD46 mAb crosslinking increased the number of GFP-LC3 (autophagosomes) puncta per cell (Fig. 3A) in epithelial cells from the asthmatic patients and controls. Quantification of GFP-LC3 puncta per cell was assayed, and the statistical data are shown in Fig. 3B. We monitored autophagy following the conversion of LC3-I to LC3-II by Western blot of the epithelial cell lysates. The presence of CD46 resulted in an increase in LC3-II in cell lysates compared to IgG control cells (Fig. 3C). The statistical data of experiments with 30 paired samples are shown in Fig. 3D (p < 0.05). To determine whether autophagosome detection subsequent to CD46 engagement was the result of an increase in autophagic flux or an accumulation of basal autophagosomes, we analyzed autophagy in the presence of lysosomal inhibitors with bafilomycin. We found that crosslinking CD46 mAb induced autophagy marker with LC3-II (*p* < 0.05) during bafilomycin clamp in primary nasal epithelial cells from the asthmatic patients as shown in Fig. 3E,F.

### Crosslinking CD46 Antibody Mediated Autophagy Against Hydrogen Peroxide-induced Apoptosis in Normal and Asthmatic Epithelial Cells

**Figure 4:**
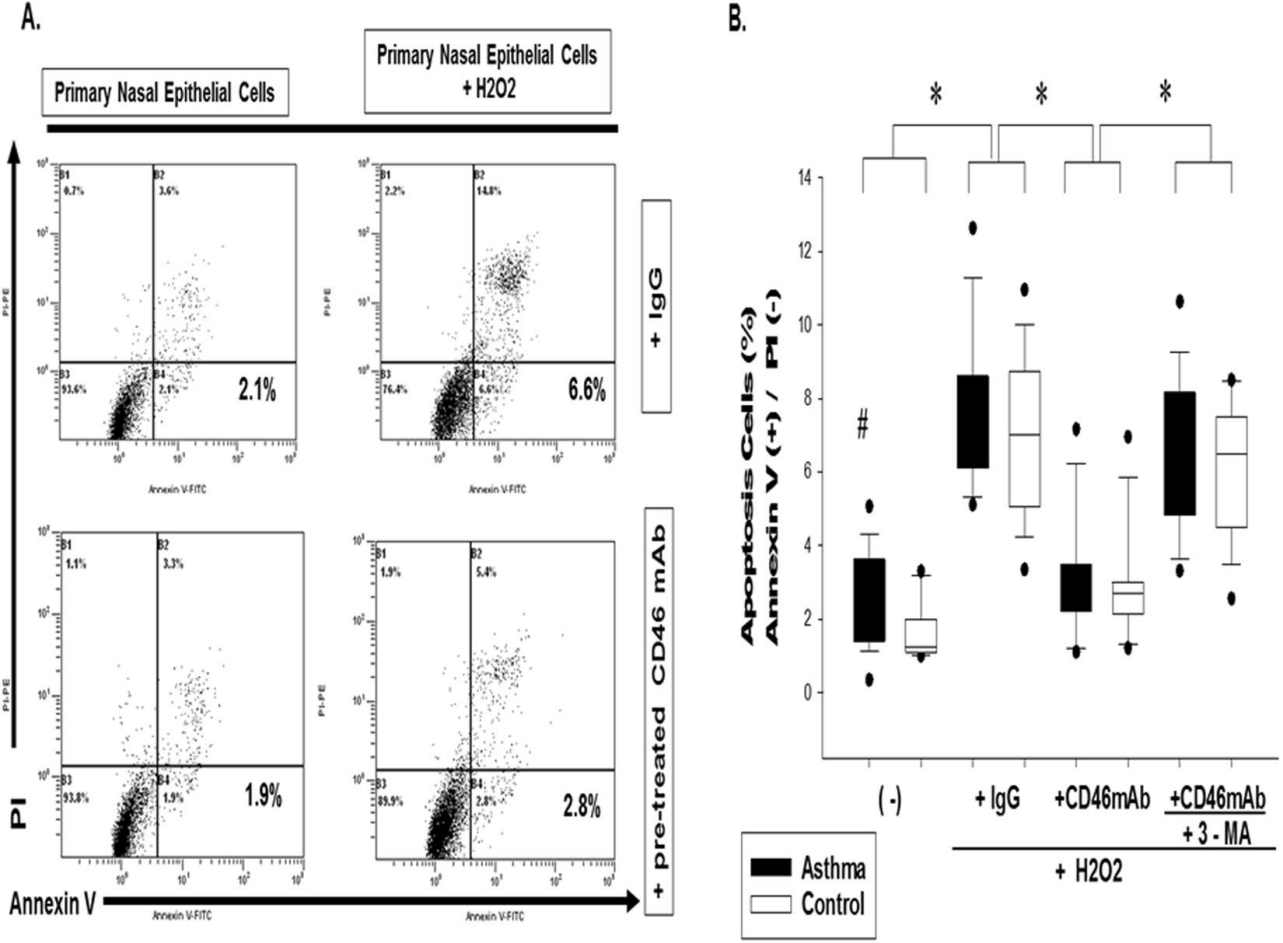
CD46 induced autophagy against H2O2-induced airway epithelial cell apoptosis in the controls and asthmatic patients. (**A**) To detect the role of CD46-induced autophagy in early apoptotic cells with Annexin V-positive but PI-negative primary nasal epithelium cells after exposure to hydrogen peroxide, a FITC Annexin V/propidium iodide Apoptosis Detection Kit I (BD Pharmingen, USA) was used. CD46 mAb (5 μg/ml) and/or autophagy inhibitor 3-methyladenine (3-MA) (10 mmol/L) (Sigma-Aldrich, St. Louis, MO) was co-incubated with primary nasal epithelium cells (1 × 10^5^ cells) from the asthmatic patients and then cultured with H2O2 (0.5 mM) for 1 hour followed by 8 hours recovery. Representative figures are shown. (**B**) Statistical data of the experiments with 30 paired samples. The Kruskal-Wallis test was used to determine significant differences. *p < 0.05 after treatment and ^#^*p* < 0.05 compared to the control group.

To evaluate the protective role of CD46-induced autophagy in the prevention of respiratory epithelium apoptosis in asthma, we evaluated hydrogen peroxide-induced epithelial cell death from CD46 mAb co-cultured with the autophagy inhibitor 3-methyladenine (3-MA). In analysis of the hydrogen peroxide-mediated cells with annexin V expression, the percentage of apoptosis of CD46 mAb-pretreated primary nasal epithelium cells from the asthmatic patients was decreased compared with the IgG control group in asthmatic patients (3.3 ± 1.8% vs. 7.3 ± 2.2%, *p* < 0.05) (Fig. 4A,B). The extent of cell death was further increased when CD46-induced autophagy was inhibited with 3-MA treatment (3.3 ± 1.8% vs. 6.4 ± 2.0%, *p* < 0.05). Experiments were performed with 30 paired samples, and the statistical data are shown in Fig. 4B.

### CD46 Antibody Inhibited 8-OHdG, IL-1β and IL-6 from Hydrogen Peroxide-induced Epithelial Cells

**Figure 5:**
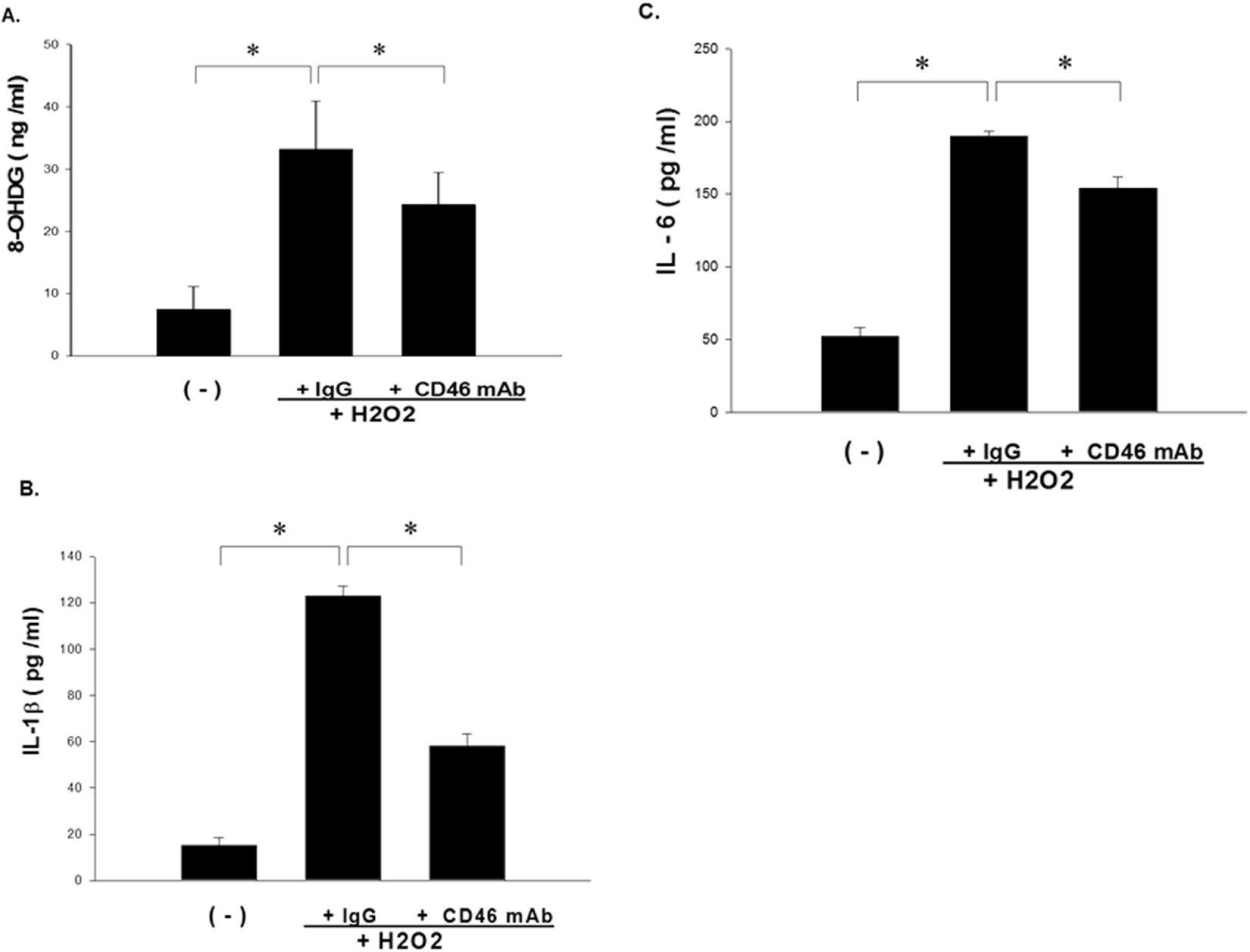
CD46 inhibited IL-1β and IL-6 from H2O2-activated airway epithelial cells from the asthmatic patients. Supernatant from anti-CD46 mAb (5 μg/ml)-pretreated primary nasal epithelium cells from the asthmatic patients after exposure to H2O2 (0.5 mM) was analyzed using 8-OHdG (**A**) and IL-1β (**B**) and IL-6 (**C**) ELISA-based systems. The Kruskal-Wallis test was used. Statistical data of the experiments with 30 paired samples are shown. *p < 0.05.

The levels of 8-OHdG, IL-1β and IL-6 in the supernatant from CD46 mAb-pretreated primary nasal epithelium cells from the asthmatic patients after exposure to H2O2 were analyzed using ELISA. The results showed decreased levels of 8-OHdG (Fig. 5A), IL-1β (Fig. 5B) and IL-6 (Fig. 5C) compared with the IgG control group (*p* < 0.05).

### Silencing *ATG5* Decreased CD46-activated Autophagy in A549 Cells

**Figure 6:**
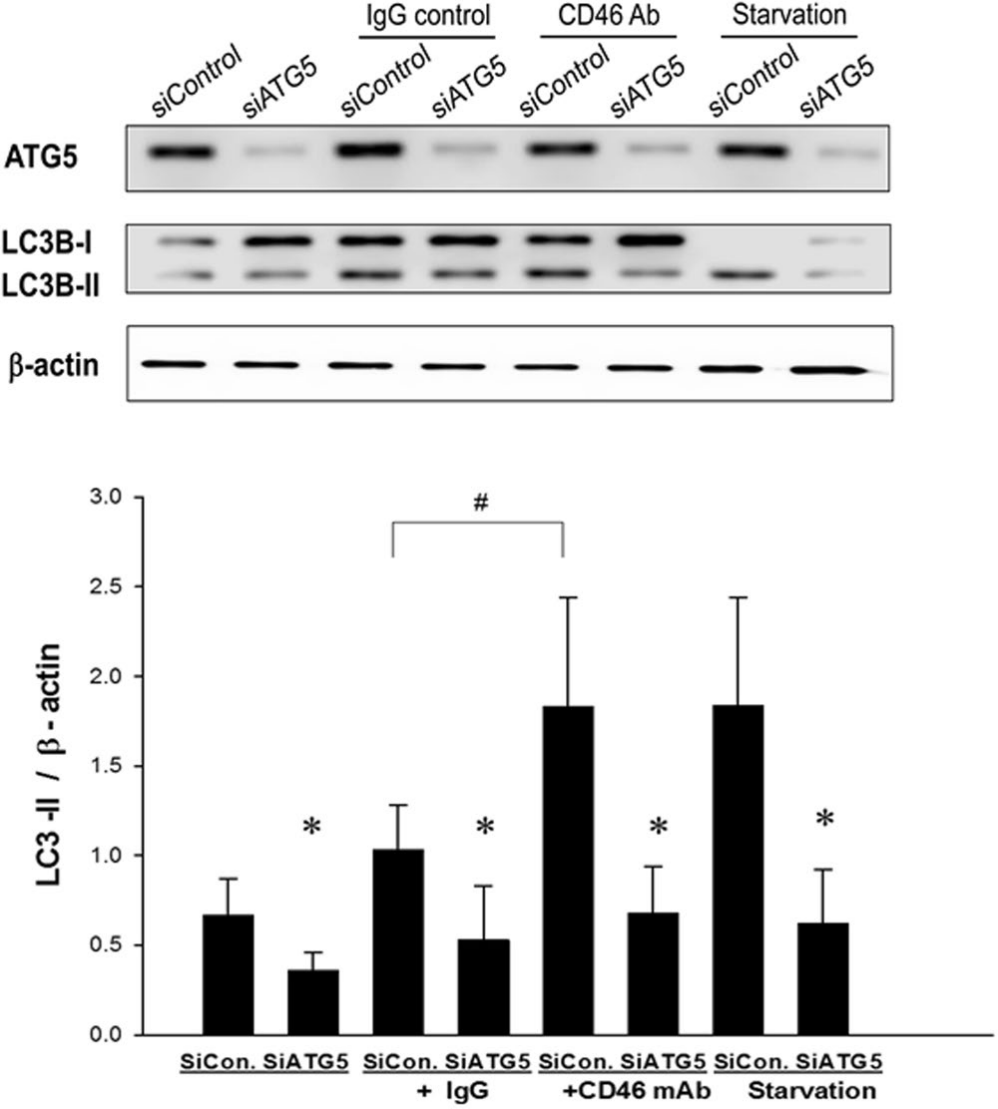
Silencing ATG5 decreased CD46-activated autophagy in A549 cells. A549 cells were transfected with small interfering RNAs (siRNA) targeting human ATG5 or control siRNA. The A549 cells were incubated for 4 hours in complete medium in the presence of anti-CD46 mAb (5 μg/ml), isotype control antibody (IgG), or in nutrient-deprived media (starvation). Proteins were extracted from the A549 cells and analyzed by Western blot using anti-ATG5 mAb, anti-LCB3-1 mAb, anti- anti-LCB3-2 mAb. The Kruskal-Wallis test was used to determine significant differences. Statistical data of the experiments are shown. *p < 0.05 after siRNA and ^#^*p* < 0.05 compared to the IgG group.

A549 cells were transfected with siRNA targeting human *ATG5* or control siRNA and incubated with anti-CD46 mAb, isotype control antibody (IgG), or in nutrient-deprived media (starvation). Proteins were extracted from the A549 human lung epithelial cells and analyzed by Western blotting (Fig. 6). The reduced expression of the *ATG5* gene for autophagy using short siRNA prevented CD46-induced and starvation-induced autophagy with LC3-II (Fig. 6).

### Crosslinking CD46 Antibody Enhanced GOPC and Inhibited PRO-IL-1β and NLRP3 Expressions from H2O2-activated A549 Cells

**Figure 7:**
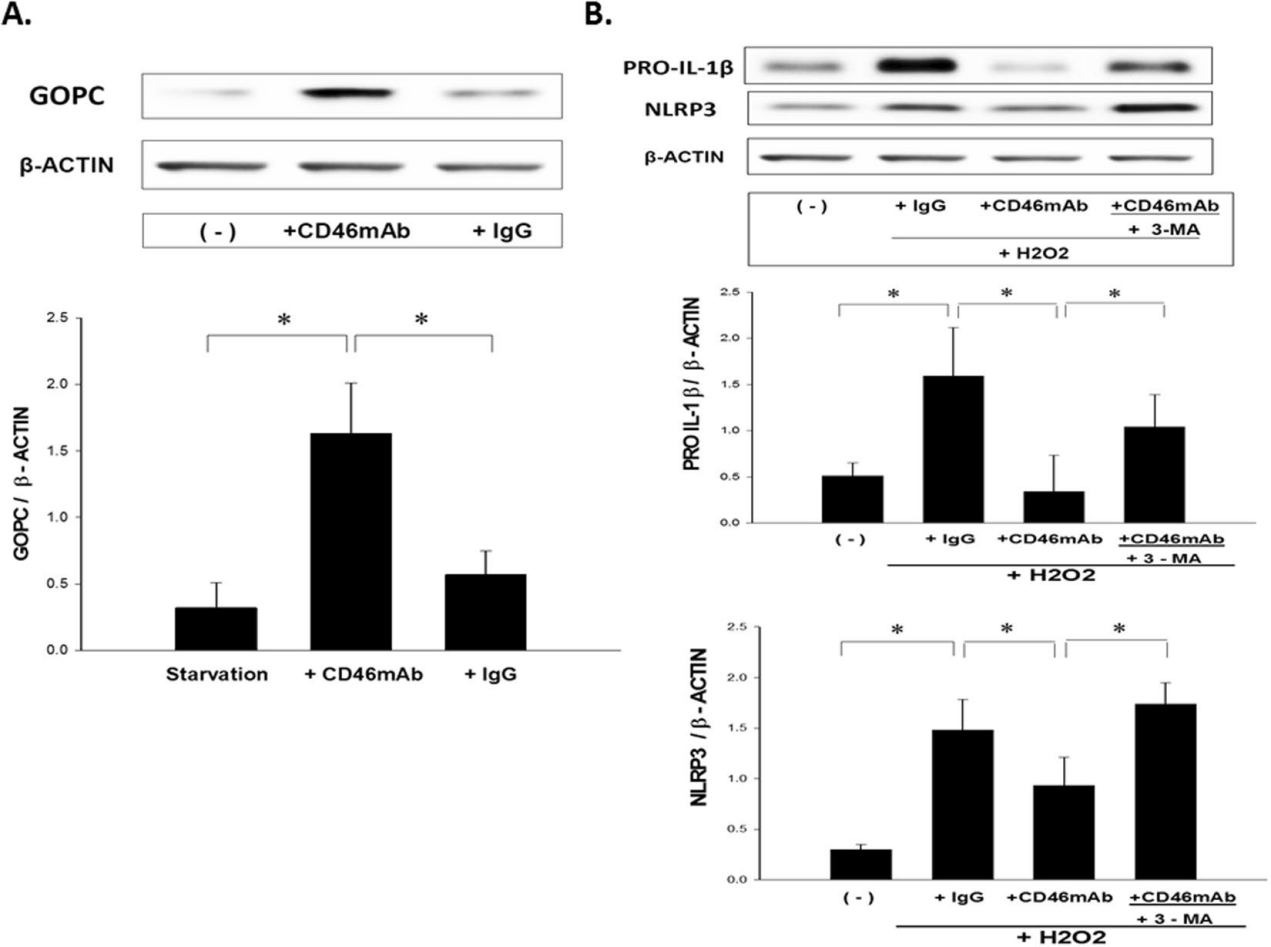
CD46 enhanced the expression of GOPC and inhibited the expressions of PRO-IL-1β and NLRP3 in H2O2-activated A549 cells. (**A**) A549 cells were incubated for 4 hours in complete medium in the presence of anti-CD46 mAb (5 μg/ml), or isotype control antibody (IgG). Immunoblotting of anti-GOPC mAb was used to analyze the GOPC expression. Statistical data of experiments on six paired samples are shown. *p < 0.05 (**B**) The anti-CD46 mAb (5 μg/ml)-pretreated A549 cells after exposure to H2O2 (0.2 mM) and cultured with the autophagy inhibitor 3-methyladenine (3-MA) (10 mmol/L) were analyzed by Western blot using anti-IL-1β mAb and anti-NLRP3 mAb. The Kruskal-Wallis test was used to determine significant differences. Statistical data of experiments with six paired samples are shown. *p < 0.05.

To evaluate the mechanism of CD46-induced autophagy in the respiratory epithelium, we examined whether CD46 crosslinking by mAb could induce induction of the scaffold protein GOPC. We demonstrated that crosslinking of CD46 by the specific mAb could induce the expression of GOPC in human lung epithelial A549 cells (*p* < 0.05) (Fig. 7A). To investigate the mechanism by which CD46-induced autophagy reduced IL-1β, we evaluated the expressions of pro-IL-1β and NLRP3 inflammasome in an H2O2-induced epithelial cell model in the presence of anti-CD46 mAb and/or the autophagy inhibitor 3-MA. The expressions of PRO-IL1β and NLRP3 in the H2O2-activated A549 cells were reduced in the presence of anti-CD46 mAb, and 3-MA treatment reversed this effect (*p* < 0.05) as shown in Fig. 7B.

## Discussion

The results of the present study showed a significantly increased surface expression of the complement regulatory protein CD46 in nasal epithelium from asthmatic patients compared to healthy subjects. In addition, there was an increase in apoptosis with a decrease in CD46 expression in fragile epithelium from the asthmatic patients, and a decrease in the expression of CD46 and increase in the expression of cleaved CASPASE-3A in Der p 2-cultured primary epithelial cells. CD46 crosslinking could induce the formation of autophagosomes and LC3-II expression in primary respiratory epithelium cells. Furthermore, CD46 mAb suppressed hydrogen peroxide-induced epithelial cell apoptosis, and treatment with the autophagy inhibitor 3-MA reversed this effect. CD46 also decreased the hydrogen peroxide-induced production of 8-OHdG, IL-1β and IL-6 from the epithelial cells. In addition, silencing ATG5 in A549 cells decreased CD46-activated autophagy with LC3-II expressions. Taken together, these results suggest that CD46 could induce autophagy and decrease oxidative stress-mediated apoptosis in respiratory epithelium, and this may offer a new potential therapeutic strategy to treat allergic asthma.

Numerous studies have reported that ROS can increase airway inflammation, and that modification of airway oxidative stress may affect the pathological features of asthma^3–13^. It has also been reported that asthmatic airway epithelial cells are susceptible to oxidative stress, and that cumulative oxidative damage contributes to apoptosis in epithelium lining asthmatic airways^16^. Complement has emerged as an important factor in the pathophysiology of asthma, and the identification of complement split products at the airway surface has been shown to be a common pathway for the induction of Th2-mediated inflammatory responses^29,30^. CD46 acts as ‘don’t-eat me’ signal and is down-regulated during apoptosis, and this leaves cells less protected against complement activation by oxidative stress^33,41–43^. It is known that respiratory epithelial cells express membrane-bound complement regulatory protein (CD46) to prevent complement-mediated autologous tissue damage. Mahtout *et al*. reported that *Porphyromonas gingivalis*, a major etiological agent of chronic periodontitis, causes shedding of CD46 expressed by epithelial cells^44^. The allergen *Dermatophagoides pteronyssinus* 2 contains a cysteine protease, and it may cause proteolysis of CD46 with a subsequent increase in the apoptosis of respiratory epithelial cells.

Studies on bronchial epithelial cells in asthmatic patients are hampered by difficulties in obtaining suitable human samples. The concept of a “united airway disease” has been proposed between allergic rhinitis and asthma. Nasal epithelial cells have been used as surrogates for lower airway cells in which nasal and bronchial cells have a similar morphological appearance and uniform cytokine expressions^45^. Varsano *et al*. demonstrated that the normal human respiratory tract from the nose to the alveoli expresses CD46, and that this expression was increased during inflammation and retained under cell culture conditions^46^. We also found that the expression of CD46 increased in asthmatic respiratory epithelium. In addition, we also detected apoptosis of the fragile respiratory epithelium concurrently with a decrease in CD46 expression. Furthermore, primary nasal epithelial cells were susceptible to mite allergen-induced apoptosis following a decrease in CD46 expression. This suggests that CD46-inhibited oxidant-induced apoptosis may offer a new strategy to treat lung injuries linked to oxidative stress in asthma.

Many studies have established an important crosslink between oxidative stress and autophagy in the pathogenic processes of asthma^26,27^. Poon *et al*. reported an increase in autophagy in human bronchial tissues of patients with asthma, and suggested an association between autophagy and reduced lung function in patients with moderately severe asthma^47^. In addition, an experimental mice model showed that autophagy protein-deficient bronchial epithelial cells were hyperresponsive to methacholine exposure, and that this contributed to smooth muscle hyperreactivity^48^. However, to date, no studies have documented the autophagy regulation of respiratory epithelium cells by CD46 stimulation. Importantly, we found that CD46 mAb could induce the formation of autophagosomes and LC3-II expression in primary respiratory epithelium cells. It is therefore reasonable to assume that increased autophagy would be necessary for epithelial apoptosis. Our results showed that hydrogen peroxide promoted epithelial apoptosis and that this was blocked by anti-CD46 mAb. In addition, we showed that treatment with the autophagy inhibitor 3-MA decreased the anti-apoptosis effects. These findings may help to explain the survival mechanisms by which CD46-activated autophagy plays a key role in the respiratory homeostatic mechanism that facilitates immune tolerance and establishment of respiratory tract integrity.

Several studies have reported that *ATG5* gene polymorphisms are associated with childhood asthma^28,49^, and that the expression of the *ATG5* gene is increased during acute asthma exacerbations in nasal epithelial cells^49^. Joubert *et al*. first demonstrated that crosslinking CD46 agonist antibodies (mAb) can directly trigger autophagy through ATG5^39^. The molecular pathway by which CD46 induces de novo formation of autophagosomes relies on the scaffold protein GOPC^39^. We further demonstrated that CD46 crosslinking by agonist antibodies induced the expression of GOPC in human lung epithelial A549 cells. Furthermore, we found that silencing *ATG5* in human lung epithelial A549 cells decreased CD46-activated autophagy with LC3-II. Therefore, we suggest that ATG5 plays an important role in the ability of respiratory epithelium to increase autophagy and protect against asthma.

During epithelial injury caused by oxidative stress in asthma, the epithelium becomes an important source of inflammatory cytokines that contribute to ongoing inflammation and airway remodeling^13,50^. Autophagy can directly regulate the secretion of cytokines, and disruption of normal autophagy pathways by ROS has been linked with increased secretion of the proinflammatory cytokine IL-1β^51,52^. Harris *et al*. demonstrated that autophagy controls IL-1β production through at least two separate mechanisms: by decreasing activation of the NLRP3 inflammasome, and by regulating PRO-IL-1β for lysosomal degradation^50^. We found that CD46 could decrease H2O2-induced oxidative stress through 8-OHdG and the production of the inflammatory cytokines IL-1β and IL-6 from epithelial cells. We further demonstrated that CD46-induced autophagy inhibited IL-1β by H2O2-activated airway epithelial cells due to a decrease in the expressions of PRO-IL-1β and NLRP3. Therefore, CD46 may be effective in ameliorating asthmatic airway diseases through modulating the autophagy signaling pathway.

In conclusion, we found that CD46 was a target antagonizing the apoptosis of oxidative stress-associated respiratory epithelial cells through autophagy. Further studies are needed to clarify the effect of autophagy in asthma and the effect of modulating CD46 on lower airway inflammation. These findings may provide further evidence regarding the practical application of CD46 in clinical practice as a treatment for asthma.

## Materials and Methods

### Subjects

A total of 60 adult patients with mild intermittent asthma with concomitant rhinitis and sensitivity to house dust mites (Der p) as proven by an IgE specific test result greater than third grade (>3.5 kU/L) using a CAP system (Pharmacia, Uppsala, Sweden) who were referred for turbinectomy were enrolled in this study. The asthmatic patients in our study were initially diagnosed at the study hospital. Definition of mild asthma was based on symptoms and a forced expiratory volume in 1 s (FEV1) of ≥80% according to the Global Initiative for Asthma guidelines. Surgery was performed under strict clinical indications, and all patients had difficulty in nose breathing. The samples obtained from the asthmatic patients were randomly subdivided into different treatment groups for immunohistochemistry, confocal immunofluorescence, flow cytometry and Western blot analysis. Healthy controls (n = 30) with normal serum IgE levels who visited the hospital for reasons unrelated to the study were enrolled as controls. Patients with either congenital or acquired immune deficiency and who were receiving systemic immune suppressive therapy were excluded. Nasal and inhaled corticosteroid treatment was stopped 4 weeks before surgery. Informed consent was obtained from each subject before participating in the study. The study was approved by the Institutional Review Board (No:101103) of Changhua Christian Hospital, and all methods were performed in accordance with the relevant guidelines and regulations.

### Tissue Sample, Cells, Antibodies and Reagents

Nasal biopsy specimens were cut into small pieces and placed in trypsin (0.025%)/EDTA (0.01%; Gibco, Grand Island, NY) for 3 hours at 37 °C and 5% CO_2_. Cells were strained through 70-mm nylon mesh (Becton Dickinson Labware, Franklin Lakes, NJ), washed, seeded at a density of 4 × 10^3^ cells/cm^2^ in T-75 cell culture flasks (Hyclone, GE Healthcare, USA), and cultured in bronchial epithelial cell medium (Hyclone) supplemented with 100 U/mL penicillin, 100 mg/mL streptomycin, and 0.25 mg/mL amphotericin B (Gibco) in a humidified atmosphere containing 5% CO_2_ at 37 °C. Confluent monolayer primary nasal epithelial cells or A549 cells (human bronchial epithelial cell line, American Type Culture Collection, Rockville, MD) were cultured with or without anti-human CD46 mAb (5 ug/ml) (Clone: MEM-258) (GeneTex, San Antonio, Texas, USA) in RPMI-1640 medium containing 10% fetal bovine serum (Gibco). Recombinant *Dermatophagoides pteronyssinus* 2 (Der p 2) (Indoor Biotechnologies, Cardiff, UK) served as the allergen. The following antibodies were used for Western blot analysis: anti-human CD46 mAb (Genetex), anti-cleaved CASPASE 3 A (Genetex), anti-ATG5 mAb (Genetex), anti-LCB3-I mAb (Genetex), anti-LCB3-II mAb (Novus Biologicals, Littleton, CO), anti-GOPC mAb (Genetex), anti-IL-1β mAb (Abcam, Cambridge, MA), anti-NLRP3 mAb (Genetex), IgG (Abcam) and β-ACTIN (Abcam).

### Histology and Immunohistochemistry

Paraffin-embedded nasal tissue samples were soaked in xylene and then sequentially in solutions of 100%, 95%, and 70% ethanol to remove the paraffin wax and for rehydration. Antigen unmasking was performed by heating the slides in retrieval buffer, and then cooled to room temperature. H2O2 block (Lab Vision, Fremont, CA) and protein block (Lab Vision, Fremont, CA) were then applied to the tissues to prevent non-specific protein binding and to block endogenous peroxidases. Rabbit monoclonal anti-human CD46 antibody (GeneTex) was diluted 1:500 with antibody diluent (Lab Vision, Fremont, CA) and applied to the tissues for 30 minutes at room temperature. Immunohistochemical staining was performed using an UltraVision Quanto Detection System HRP (Thermo Fisher Scientific, Waltham, MA). Visualization was achieved using the diaminobenzidine (DAB) method. Slides were counterstained with hematoxylin.

### Confocal Immunofluorescence

Cells were incubated with diluted Autophagy Reagent A according to the manufacturer’s recommendations in a FlowCellect™ Autophagy LC3 Antibody-based Assay Kit (Millipore, Billerica, MA). This kit contains two key detection reagents to help facilitate the monitoring of lipidated LC3-II in a given cell system. Briefly, the use of selective permeabilization solution discriminates between cytosolic LC3 from autophagic LC3 by extracting the soluble cytosolic proteins, while protecting LC3 which has been sequestered into the autophagosome. Primary nasal epithelial cells were incubated with Reagent A at 37 °C and 5% CO_2_ for 60 min. Cells were washed and resuspended in 100 μL of Reagent B, centrifuged immediately, and resuspended in 100 μL of Assay buffer with 1:20 diluted FITC-conjugated anti-LC3 antibody. LC3-II was photographed live on a Confocal Olympus FV1200 fluorescent microscope. The number of GFP-LC3 vesicles in primary nasal epithelial cells was calculated from 200 cells for each experiment. Quantification of GFP-LC3 puncta per cell was assayed. For terminal nucleotidyl transferase-mediated nick end labeling (TUNEL) assay, we used an ApopTag Plus Peroxidase *In Situ* Apoptosis Detection Kit (Takara, Shiga, Japan) according to the manufacturer’s instructions. In order to detect apoptosis of CD46-expressing cells, confocal microscopic analyses between intact and fragile primary nasal epithelium with TUNEL (FITC), CD46 (PE), and 4′,6-diamidino-2-phenylindole (DAPI) (nuclear stain) were performed. To evaluate whether CD46 crosslinking could induce autophagy in primary upper airway epithelial cells, epithelial cells were incubated for 4 hours in complete medium either in the presence of anti-CD46 mAb (5 μg/ml), isotype control antibody (IgG), or in nutrient-deprived media (starvation), and quantification of GFP-LC3 puncta (autophagosomes) in the nasal epithelial cells was performed using confocal microscopy.

### Flow Cytometry and Annexin V/Propidium Iodide Double Staining

To detect the role of CD46-induced autophagy in primary upper respiratory epithelial cells after exposure to hydrogen peroxide, an FITC Annexin V/propidium iodide Apoptosis Detection Kit I (BD Pharmingen, USA) was used. Anti-CD46 mAb (5 μg/ml) and/or autophagy inhibitor 3-methyladenine (3-MA) (10 mmol/L) (Sigma-Aldrich, St. Louis, MO) was co-incubated with primary nasal epithelial cells (1 × 10^5^ cells) and then cultured with H2O2 (0.5 mM) for 1 hour, followed by 8 hours recovery. The treated cells were then stained with propidium iodide and Annexin V-FITC for 15 minutes according to the manufacturer’s instructions, and then subjected to flow cytometry analysis (FC500, Beckman Coulter, Fullerton, CA).

### Enzyme-Linked Immunosorbent Assay (ELISA)

The concentrations of IL-1β and IL-6 in cell supernatants were determined using a commercially available ELISA-based assay system (R&D Systems, London, UK). The expression of the oxidative stress marker 8-hydroxy-2′-deoxyguanosine (8-OHdG) induced by oxygen radicals was measured using a highly sensitive 8-OHdG ELISA kit (JalCA, Fukuroi, Shizuoka, Japan). Supernatant from anti-CD46 mAb (5 μg/ml) pretreated primary nasal epithelium cells from the asthmatic patients after exposure to H2O2 (0.5 mM) were analyzed with 8-OHdG and IL-1β and IL-6 ELISA-based systems.

### Gene Silencing

The mechanism of the CD46 mAb-mediated de-novo formation of autophagosomes has been shown to be regulated by *ATG5* gene-induced autophagy^39^. Small interfering RNAs (siRNAs) targeting human *ATG5* (Sense: (5′->3′) GAACCAUACUAUUUGCUUUtt; and Antisense: AAAGCAAAUAGUAUGGUUCtg) or control siRNA (GeneDirex) were purchased from Cell Signaling Technology. Cells were transfected using the siRNA transfection reagent Lipofectamine™ RNAiMAX according to the manufacturer’s instructions (Invitrogen, Carlsbad, CA). Diluted siRNA (final = 20 nM) added in 2 ml Opti-MEM I Medium (Invitrogen, Carlsbad, CA) without serum in the 100 mm culture plate. Mixed gently (30 µl Lipofectamine™ RNAiMAX to each well containing the diluted siRNA molecules) and incubate for 20 minutes at room temperature. A549 cells (1 × 10^5^ cells/mL) solution in antibiotic-free complete medium and mixed with siRNA- Lipofectamine™ RNAiMAX complexes after 24 hours, replace the transfection medium with complete medium and continue incubation 72 hours for assay.

### Western Blot Analysis

Protein levels of CD46, cleaved CASPASE 3A, ATG5, LCB3-I and LCB3-II, GOPC, PRO-IL-1β and NLRP3 were determined by Western blot analysis. Equal amounts of proteins in each study groups were ascertained using a Bio-Rad protein assay kit (Bio-Rad, Hercules, CA). Cellular proteins were resolved by 10% SDS-polyacrylamide gel. After electrophoresis, protein levels were determined by Western blot analysis.

### Statistical analysis

All data were presented as mean ± SD. As the continuous variables were not in normal distribution, nonparametric statistics including the Wilcoxon rank-sum test was used for comparisons. Groups of datasets in each treatment group were compared using the Kruskal-Wallis test, followed by the Duncan test. A *p* value less than 0.05 was considered to be statistically significant.

## Electronic supplementary material

Supplement figures

## References

[1] Ijaz T (2014). Systems biology approaches to understanding Epithelial Mesenchymal Transition (EMT) in mucosal remodeling and signaling in asthma. World Allergy Organ J..

[2] Cardinale F, Giordano P, Chinellato I, Tesse R (2013). Respiratory epithelial imbalances in asthma pathophysiology. Allergy Asthma Proc..

[3] Auerbach A, Hernandez ML (2012). The effect of environmental oxidative stress on airway inflammation. Curr. Opin. Allergy Clin. Immunol..

[4] Chung KF, Marwick JA (2010). Molecular mechanisms of oxidative stress in airways and lungs with reference to asthma and chronic obstructive pulmonary disease. Ann N.Y. Acad. Sci..

[5] Fitzpatrick AM, Park Y, Brown LA, Jones DP (2014). Children with severe asthma have unique oxidative stress-associated metabolomic profiles. J Allergy Clin. Immunol..

[6] Fatani SH (2014). Biomarkers of oxidative stress in acute and chronic bronchial asthma. J Asthma..

[7] Moreno-Macias H, Romieu I (2014). Effects of antioxidant supplements and nutrients on patients with asthma and allergies. J Allergy Clin Immunol..

[8] Jiang L (2014). Molecular characterization of redox mechanisms in allergic asthma. Ann Allergy Asthma Immunol..

[9] Zuo L, Otenbaker NP, Rose BA, Salisbury KS (2013). Molecular mechanisms of reactive oxygen species-related pulmonary inflammation and asthma. Mol. Immunol..

[10] Boldogh I (2005). ROS generated by pollen NADPH oxidase provide a signal that augments antigen-induced allergic airway inflammation. J Clin. Invest..

[11] Celik M (2012). Oxidative stress in the airways of children with asthma and allergic rhinitis. Pediatr Allergy Immunol..

[12] Fitzpatrick AM, Brown LA, Holguin F, Teague WG (2009). National Institutes of Health/National Heart Lung, and Blood Institute Severe Asthma Research Program. Levels of nitric oxide oxidation products are increased in the epithelial lining fluid of children with persistent asthma. J Allergy Clin. Immunol..

[13] Brown SD (2012). Airway TGF-beta1 and oxidant stress in children with severe asthma: association with airflow limitation. J Allergy Clin. Immunol..

[14] Zalewski PD, Ruffin RE (2008). Apoptosis-regulatory factors as potential drug targets in the epithelium of normal and inflamed airways. Curr. Mol. Pharmacol..

[15] Xiao C (2011). Defective epithelial barrier function in asthma. J Allergy Clin. Immunol..

[16] Bucchieri F (2002). Asthmatic bronchial epithelium is more susceptible to oxidant-induced apoptosis. Am J Respir. Cell Mol. Biol..

[17] Ryter SW, Cloonan SM, Choi AM (2013). Autophagy: a critical regulator of cellular metabolism and homeostasis. Mol Cells..

[18] Navarro-Yepes J (2014). Oxidative stress, redox signaling, and autophagy: cell death versus survival. Antioxid Redox Signal..

[19] Deretic V, Saitoh T, Akira S (2013). Autophagy in infection, inflammation and immunity. Nat. Rev. Immunol..

[20] Omata Y, Lim YM, Akao Y, Tsuda L (2014). Age-induced reduction of autophagy-related gene expression is associated with onset of Alzheimer’s disease. Am. J Neurodegener. Dis..

[21] Kamat PK, Kalani A, Kyles P, Tyagi SC, Tyagi N (2014). Autophagy of mitochondria: a promising therapeutic target for neurodegenerative disease. Cell Biochem Biophys..

[22] Kongara S, Karantza V (2012). The interplay between autophagy and ROS in tumorigenesis. Front Oncol..

[23] Choi AM, Ryter SW, Levine B (2013). Autophagy in human health and disease. N. Engl. J Med..

[24] Huang J, Lam GY, Brumell JH (2011). Autophagy signaling through reactive oxygen species. Antioxid Redox Signal..

[25] Dodson M, Darley-Usmar V, Zhang J (2013). Cellular metabolic and autophagic pathways: traffic control by redox signaling. Free Radic. Biol. Med..

[26] Araya J, Hara H, Kuwano K (2013). Autophagy in the pathogenesis of pulmonary disease. Intern Med..

[27] Jyothula SS, Eissa NT (2013). Autophagy and role in asthma. Curr Opin. Pulm. Med..

[28] Poon A, Eidelman D, Laprise C, Hamid Q (2012). ATG5, autophagy and lung function in asthma. Autophagy..

[29] Laumonnier Y, Schmudde I, Köhl J (2011). The role of complement in the diagnosis and management of allergic rhinitis and allergic asthma. Curr Allergy Asthma Rep..

[30] Zhang X, Ko¨h J (2010). A complex role for complement in allergic asthma. Expert Rev Clin Immunol.

[31] Christmas SE (2006). Levels of expression of complement regulatory proteins CD46, CD55 and CD59 on resting and activated human peripheral blood leucocytes. Immunology.

[32] Riley-Vargas RC (2004). CD46: expanding beyond complement regulation. Trends Immunol.

[33] Elward K (2005). CD46 plays a key role in tailoring innate immune recognition of apoptotic and necrotic cells. J Biol Chem.

[34] Kemper C (2003). Activation of human CD4+ cells with CD3 and CD46 induces a T-regulatory cell 1 phenotype. Nature.

[35] Tsai YG (2014). Enhanced CD46-induced regulatory T cells suppress allergic inflammation after Dermatophagoides pteronyssinus-specific immunotherapy. J Allergy Clin Immunol..

[36] Tsai YG (2012). Functional defects of CD46-induced regulatory T cells to suppress airway inflammation in mite allergic asthma. Laboratory Investigation.

[37] Astier AL, Meiffren G, Freeman S, Hafler DA (2006). Alterations in CD46-mediated Tr1 regulatory T cells in patients with multiple sclerosis. J Clin Invest.

[38] Grumelli S, Lu B, Peterson L, Maeno T, Gerard C (2011). CD46 protects against chronic obstructive pulmonary disease. Plos One.

[39] Joubert PE (2009). Autophagy induction by the pathogen receptor CD46. Cell Host Microbe..

[40] Meiffren G (2010). Pathogen recognition by the cell surface receptor CD46 induces autophagy. Autophagy..

[41] Wang L (2014). Nrf2 signaling modulates cigarette smoke-induced complement activation in retinal pigmented epithelial cells. Free Radic. Biol. Med..

[42] Trouw LA, Blom AM, Gasque P (2008). Role of complement and complement regulators in the removal of apoptotic cells. Mol Immunol..

[43] Vogt SD (2011). Retinal pigment epithelial expression of complement regulator CD46 is altered early in the course of geographic atrophy. Exp Eye Res..

[44] Mahtout H, Chandad F, Rojo JM, Grenier D (2009). Porphyromonas gingivalis mediates the shedding and proteolysis of complement regulatory protein CD46 expressed by oral epithelial cells. Oral Microbiol Immunol..

[45] McDougal CM (2008). Nasal epithelial cells as surrogates for bronchial epithelial cells in airway inflammation studies. Am J Respir Cell Mol Biol..

[46] Varsano S, Frolkis I, Ophir D (1995). Expression and distribution of cell-membrane complement regulatory glycoproteins along the human respiratory tract. Am. J Respir. Crit. Care. Med..

[47] Poon AH (2012). Genetic and histologic evidence for autophagy in asthma pathogenesis. J Allergy Clin. Immunol..

[48] Inoue D (2011). Inducible disruption of autophagy in the lung causes airway hyper-responsiveness. Biochem Biophys Res Commun..

[49] Martin. LJ (2012). Functional variant in the autophagy-related 5 gene promoter is associated with childhood asthma. Plos One.

[50] Takizawa H (2004). Diesel exhaust particles and their effect on induced cytokine expression in human bronchial epithelial cells. Curr Opin Allergy Clin Immunol..

[51] Crisan TO (2012). Inflammasome-independent modulation of cytokine response by autophagy in human cells. Plos One.

[52] Harris J (2011). Autophagy controls IL-1beta secretion by targeting pro-IL-1beta for degradation. J Biol Chem..

