# Supplementary material for: Complement regulatory protein CD46 induces autophagy against oxidative stress-mediated apoptosis in normal and asthmatic airway epithelium
Source: Sci Rep. 2018 Aug 28;8:12973. doi: 10.1038/s41598-018-31317-5 (PMC6113329; doi:10.1038/s41598-018-31317-5)
Supplement: Supplementary file 1 — Supplement figures [file 41598_2018_31317_MOESM1_ESM.docx]

**Supplementary Information file**

**Complement regulatory protein CD46 induces autophagy against oxidative stress-mediated apoptosis in normal and asthmatic airway epithelium**

**Yi-Giien Tsai; Yung-Sung Wen; Jiu-Yao Wang; Kuender D. Yang; Hai-Lun Sun; Jia-Hung Liou; Ching-Yuang Lin**

**
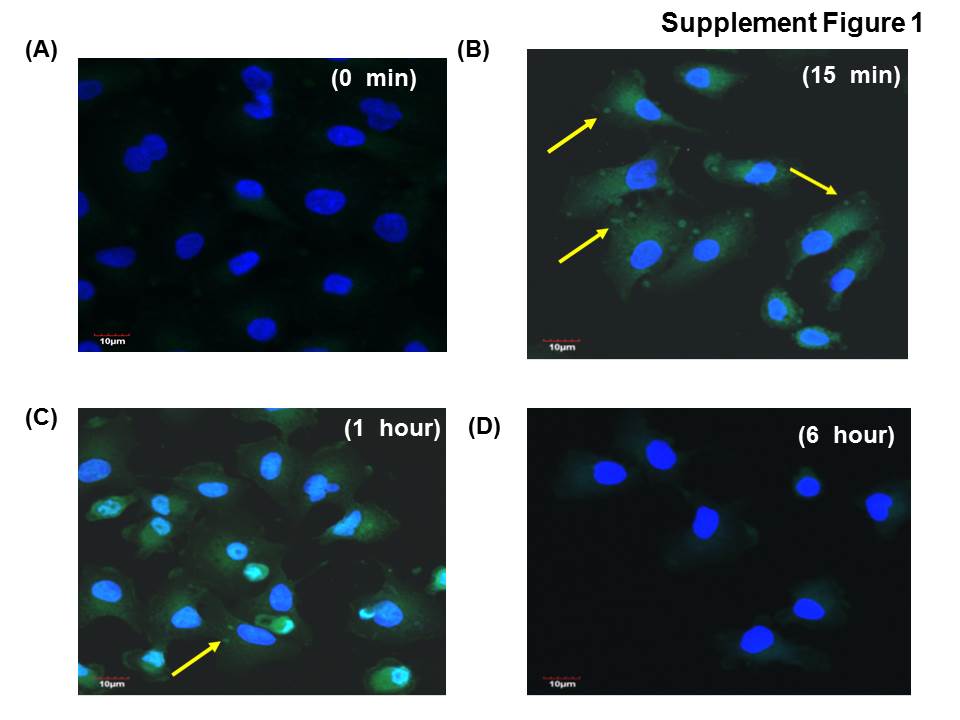
**

**Supplement Figure 1.** Crosslinking CD46 mAb at the primary nasal epithelial cells surface induces macropinocytosis-like internalization (yellow arrow), and leads to the degradation of cell surface CD46. Primary nasal epithelial cells were incubated at 37°C with (A) complete medium, (B) anti-CD46 mAb (5 μg/ml) for 15 minutes, (C) for one hour, and (D) for six hours and CD46 expression was analyzed by confocal microsopy. Representative profiles are shown (n=3) with essentially identical results.

**
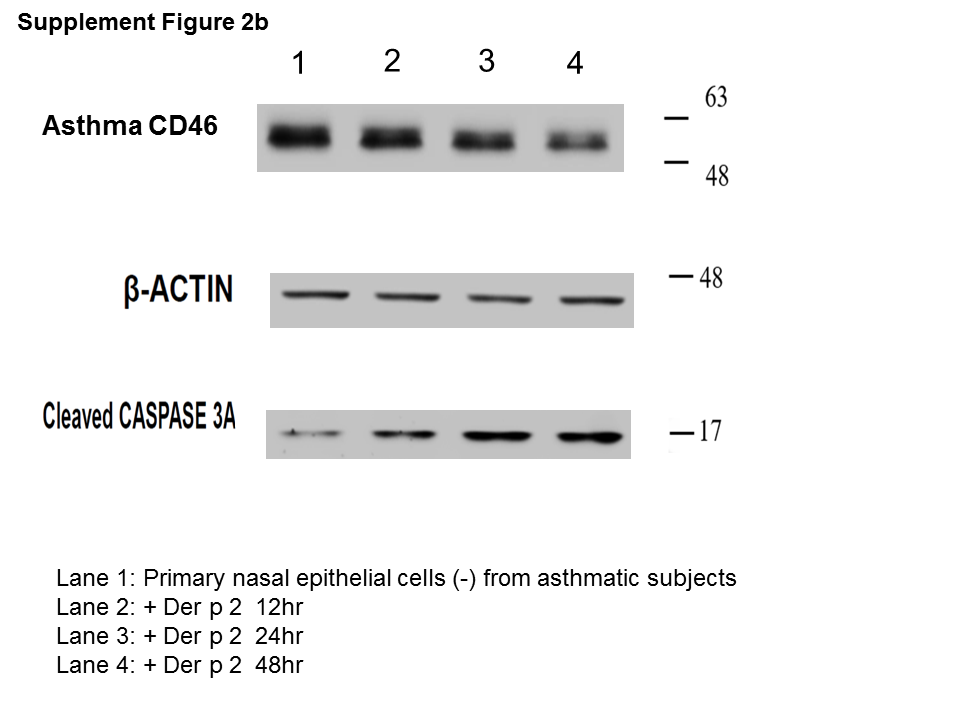
**

**
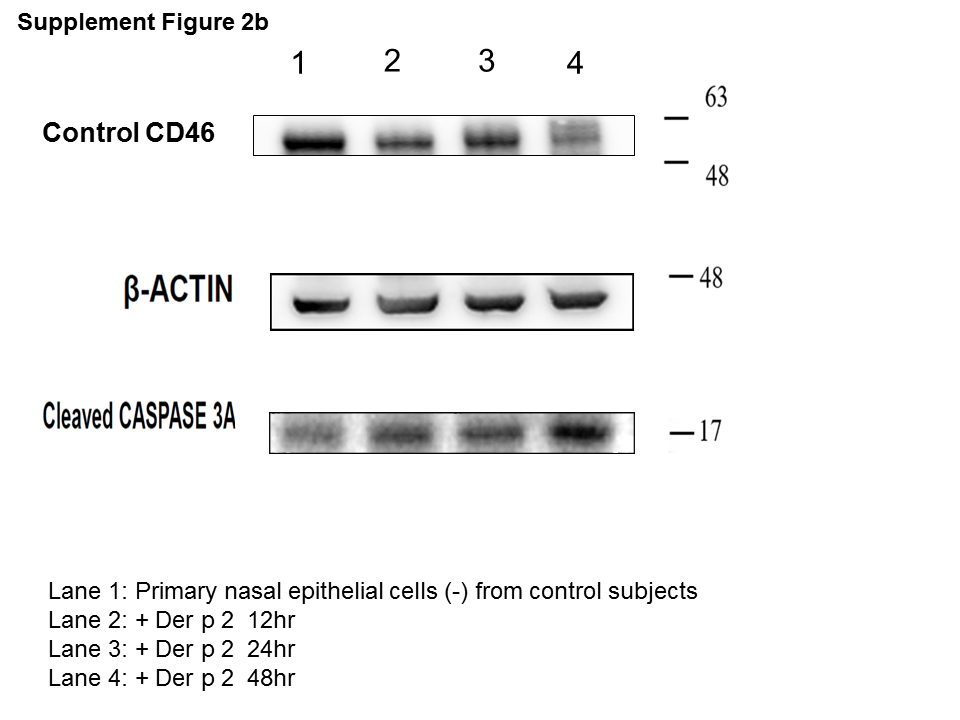
**

**
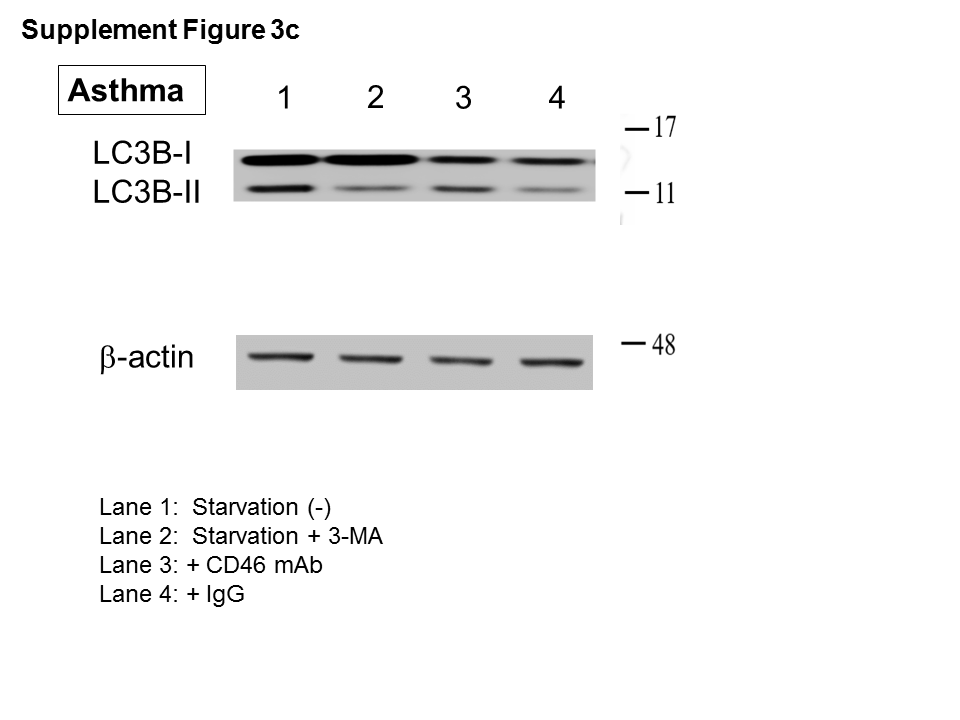
**

**
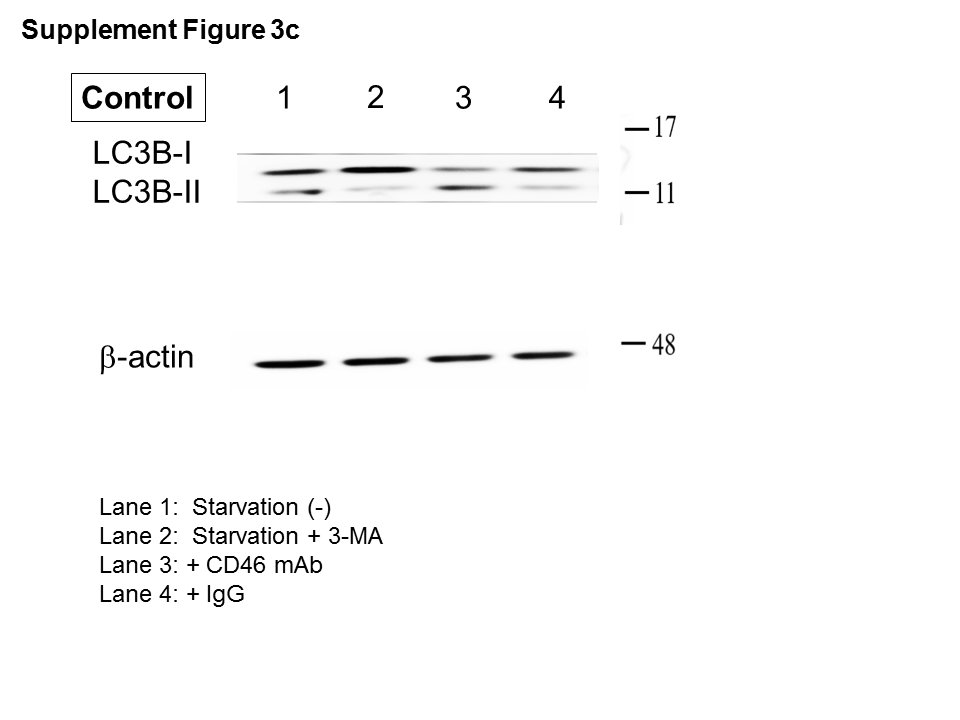
**

**
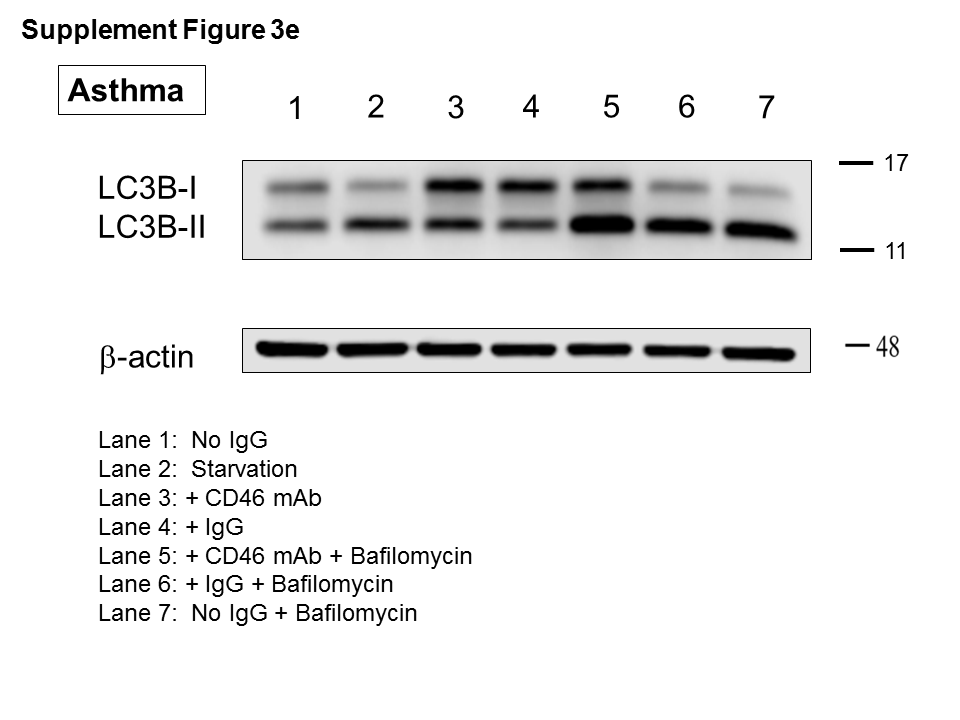
**


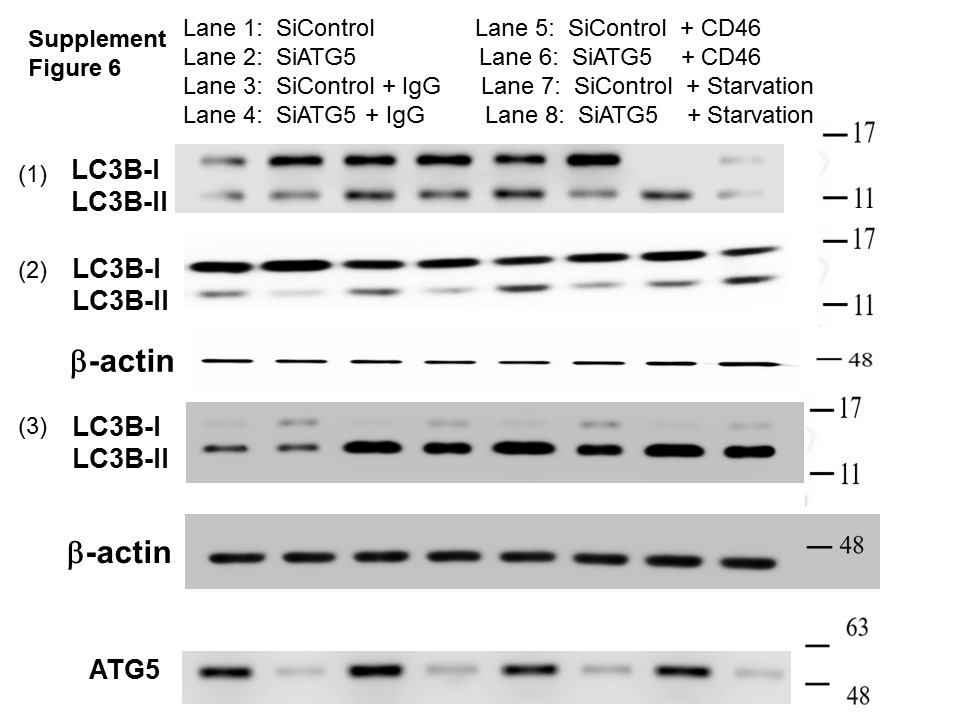


**
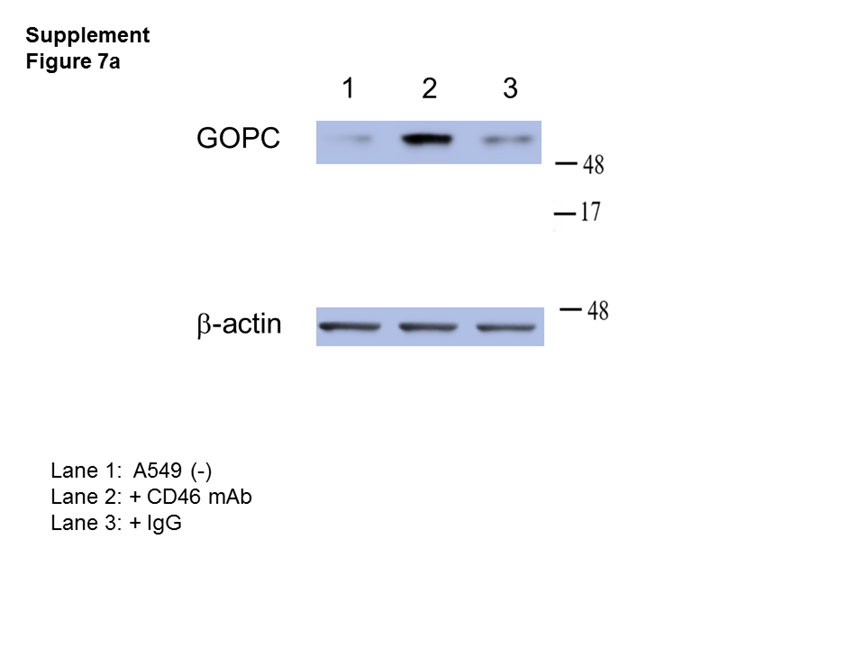
**

**
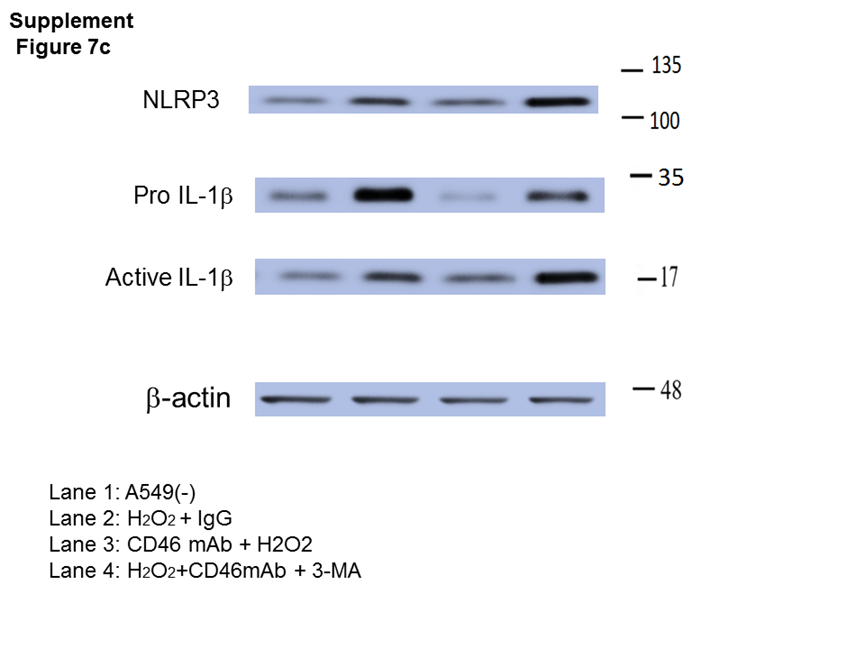
**
